# Systematic Review and Meta-Analysis of the Efficacy and Safety of Combined Epinephrine and Corticosteroid Therapy for Acute Bronchiolitis in Infants

**DOI:** 10.3389/fphar.2017.00396

**Published:** 2017-06-22

**Authors:** Kok P. Kua, Shaun W. H. Lee

**Affiliations:** ^1^School of Pharmacy, Monash University MalaysiaBandar Sunway, Malaysia; ^2^Department of Pharmacy, Petaling District Health Office, Ministry of Health MalaysiaPetaling Jaya, Malaysia

**Keywords:** bronchiolitis, epinephrine, corticosteroid, dexamethasone, respiratory syncytial virus infections, infant, meta-analysis, systematic review

## Abstract

**Objective:** To evaluate the effectiveness of combined epinephrine and corticosteroid therapy for acute bronchiolitis in infants.

**Methods:** Four electronic databases (MEDLINE, EMBASE, CINAHL, and CENTRAL) were searched from their inception to February 28, 2017 for studies involving infants aged less than 24 months with bronchiolitis which assessed the use of epinephrine and corticosteroid combination therapy. The methodological quality of the included studies was assessed using the Cochrane Collaboration's Risk of Bias Tool. A random-effects meta-analysis was used to pool the effect estimates. The primary outcomes were hospital admission rate and length of hospital stay.

**Results:** Of 1,489 citations identified, 5 randomized controlled trials involving 1,157 patients were included. All studies were of high quality and low risk of bias. Results of the meta-analysis showed no significant differences in the primary outcomes. Hospitalization rate was reduced by combinatorial therapy of epinephrine and corticosteroid in only one out of five studies, whereas pooled data indicated no benefit over epinephrine plus placebo. Clinical severity scores were significantly improved in all five RCTs when assessed individually, but no benefit was observed compared to epinephrine monotherapy when the data were pooled together. Pooled data showed that combination therapy was more effective at improving oxygen saturation level (mean difference: −0.70; 95% confidence interval: −1.17 to −0.22, *p* = 0.004). There was no difference in the risk of serious adverse events in infants treated with the combined epinephrine and corticosteroid therapy.

**Conclusions:** Combination treatment of epinephrine and dexamethasone was ineffective in reducing hospital admission and length of stay among infants with bronchiolitis.

## Introduction

Bronchiolitis is the most common lower respiratory tract infection during the first year of life (Hall et al., 2009). In the United States alone, bronchiolitis accounts for 234 thousand emergency department visits (Mansbach et al., 2005) and 140 thousand hospital admissions annually among children younger than 2 years (Hasegawa et al., 2013), with an estimated cost of $1.73 billion in 2009 (Hasegawa et al., 2013). It is manifested by extensive inflammation and edema of the airways, increased mucus production, and sloughing of airway epithelial cells (Florin et al., 2017). The classic clinical presentation of bronchiolitis begins with symptoms of a viral upper respiratory infection, such as low-grade fever, rhinorrhea, and nasal congestion that develop to the lower respiratory tract over several days (Meissner, 2016). Timing of symptom progression can vary, but a hallmark of bronchiolitis is the minute-to-minute disparity in clinical findings, as mucus and debris in the airways are cleared by coughing or as the child's condition alters from sleep to agitation (NICE, 2015). While various definitions of bronchiolitis have been proposed, the terminology is commonly applied as the first episode of wheezing in infants younger than 12 months of age (Meissner, 2016).

The variable course of bronchiolitis and the inability of physicians to predict whether supportive care is necessary frequently results in hospital admission even when symptoms are not severe. Although the clinical characteristics of bronchiolitis attributable to different viruses are usually indistinguishable, some variations in disease severity have been reported. For instance, it has been shown that rhinovirus-associated bronchiolitis may result in a shorter length of hospitalization compared with bronchiolitis that is due to respiratory syncytial virus (RSV) (Mansbach et al., 2012).

Despite the considerable burden associated with bronchiolitis, it has been challenging to determine the best possible care for a young child presented with this illness owing to the lack of curative therapy (Meissner, 2016). Beta-2 agonist bronchodilator, epinephrine, corticosteroid, hypertonic saline, supplemental oxygen, antibiotic therapy, antiviral therapy, cool mist or saline aerosol, suctioning, and chest physiotherapy are not recommended for the treatment of children with bronchiolitis (Smith et al., 2017). Clinicians may choose not to administer oxygen supplementation when oxyhemoglobin saturation exceeds 90%. Intravenous or nasogastric fluids may be utilized for children with bronchiolitis who fail to maintain hydration orally (Ralston et al., 2014). No available treatment effectively shortens the course of bronchiolitis illness or accelerates the resolution of symptoms. Treatment modality is supportive, and the vast majority of children with bronchiolitis do well irrespective of how the disease is managed. The intensity of pharmacological interventions among hospitalized children has been depicted to have diminutive relationship to the severity of illness (Willson et al., 2001; Mittal et al., 2014).

Routine use of bronchodilators is not recommended for the treatment of bronchiolitis by many guidelines (SIGN, 2006; Turner et al., 2008; Friedman et al., 2014; Ralston et al., 2014; NICE, 2015) due to the scarcity of definitive evidence. Numerous studies have evaluated the role of bronchodilator for the treatment of bronchiolitis, and systematic reviews have demonstrated no consistent benefit (Florin et al., 2017). Multiple studies have also examined the impact of corticosteroid in the management of children with bronchiolitis (Florin et al., 2017). Data from two large multicenter trials have indicated no benefit to corticosteroid monotherapy in reducing hospital admission (Corneli et al., 2007; Plint et al., 2009), and this was similarly noted in a Cochrane review (Fernandes et al., 2013).

Synergy between corticosteroid and beta-2 agonist has been well documented in clinical trials of asthma management (Greening et al., 1994; Pauwels et al., 1997; Barnes, 2007; Giembycz et al., 2008). Basic science literature has also shown that beta-2 agonist and corticosteroid enhance each other's effectiveness, especially with regard to anti-inflammatory gene expression (Kaur et al., 2008; Holden et al., 2011). Whilst several models advocate mechanisms of action for this synergy (Mak et al., 1995; Roth et al., 2002), results of *in vitro* studies of airway cells have highlighted that beta-2 adrenoceptor agonist can enhance the ability of corticosteroid to promote responses via the glucocorticoid receptor (Kaur et al., 2008). It is imperative to note that these findings reveal beta-2 adrenoceptor agonist is not only steroid-sparing, but also potentiates the maximal efficacy of the response to corticosteroid to a level that cannot be attained by corticosteroid alone (Kaur et al., 2008). This effect is purported to mimic the clinical observation in the context of asthma (Giembycz et al., 2008). In the scenario of wheezing infants and bronchiolitis, three small studies have demonstrated similar synergy between both epinephrine and dexamethasone and salbutamol and dexamethasone (Tal et al., 1983; Kuyucu et al., 2004; Bentur et al., 2005).

The encouraging findings from the Canadian Bronchiolitis Epinephrine Steroid Trial (CanBEST) suggested that the combination of nebulized epinephrine and oral dexamethasone treatment given to outpatients with bronchiolitis decreased rate of hospital admission and improved clinical symptoms (Plint et al., 2009). To date, several randomized controlled trials (RCTs) have been conducted to investigate this combination therapy, with some showing promising results (Plint et al., 2009), whilst others reporting a null effect (Bawazeer et al., 2014). In light of the contradictory results and the plausible basic and clinical evidence for a synergistic effect, in this study, we appraised all published RCTs to summarize the efficacy and safety of combined epinephrine and corticosteroid for treating bronchiolitis in young infants.

## Methods

A systematic review was undertaken in accordance with the methodological and reporting standards recommended by the PRISMA statement (Moher et al., 2009). Four databases: MEDLINE, EMBASE, CINAHL, and CENTRAL were searched from inception through February 28, 2017 using a combination of keywords (Supplementary Material). Studies were included if they were: randomized controlled trials (RCTs), involved infants aged 24 months or less diagnosed with bronchiolitis, and examined the use of epinephrine and corticosteroid combination therapy compared to standard care or any other drug intervention. Full texts of relevant articles were retrieved. Corresponding author of a study was contacted for additional data (Bentur et al., 2005). The quality of included studies was assessed using Cochrane Collaboration's Risk of Bias Tool (Higgins et al., 2011). The primary outcome measures were hospital admission rate and length of hospital stay. Secondary outcomes included: clinical severity score, heart rate, respiratory rate, oxygen saturation, and adverse event. Data were presented qualitatively. A random effects meta-analysis was performed since clinical heterogeneity was expected (Riley et al., 2011). Statistical heterogeneity across studies was measured by Cochran Q test and *I*^2^ statistic (Higgins et al., 2003). All analyses were performed using Review Manager (RevMan) software, version 5.3.

## Results

### Study selection and study characteristics

The search found 1,489 citations, of which 63 full-text articles were assessed and five RCTs were included in the current review, representing 1,157 patients with a diagnosis of bronchiolitis (Kuyucu et al., 2004; Bentur et al., 2005; Mesquita et al., 2009; Plint et al., 2009; Bawazeer et al., 2014). Overall, 330 patients were given epinephrine and corticosteroid combination therapy (28.5%) compared with 827 in the control arms (71.5%). Three studies were carried out in emergency department (Mesquita et al., 2009; Plint et al., 2009; Bawazeer et al., 2014), one in an inpatient setting (Bentur et al., 2005), and another in an outpatient clinic and emergency department (Kuyucu et al., 2004). The length of follow-up ranged from 4 h to 3 months (Table 1).

**Table 1:** Summary of main results of the included studies.

**Study, country**	**Setting**	**Population (sample size)**	**Interventions**	**Age (months)^§^**	**Timing of primary outcome assessment**	**Main findings**
Saudi Arabia (Bawazeer et al., 2014)	Pediatric emergency department in a hospital	Infants aged 1 to 12 months within 7 days of onset of respiratory symptoms and RDAI score of 5 to 15 (*N* = 162)	1. Three doses of Neb. Racemic epinephrine 5.6 mg at 0, 30, and 90 min apart + Oral Dexamethasone 1 mg/kg as loading, followed by 0.6 mg/kg once daily for 2 days (*n* = 45)	4.7 ± 2.8	4 h, 3 days, and 7 days	Hospitalization rate, RDAI score, respiratory rate, and oxygen saturation were similar across all treatment groups. At 4-h after treatment, Group 1 showed a significant improvement in heart rate over time compared to other groups (*p* = 0.04).
			2. Three doses of Neb. Salbutamol + Oral Dexamethasone 1 mg/kg as loading, followed by 0.6 mg/kg once daily for 2 days (*n* = 40)	4.6 ± 2.2		
			3. Three doses of Neb. Racemic epinephrine 5.6 mg at 0, 30, and 90 min apart + Oral Placebo for 2 days (*n* = 39)	4.2 ± 2.5		
			4. Three doses of Neb. Salbutamol + Oral Placebo for 2 days (*n* = 38)	4.9 ± 2.4		
Israel (Bentur et al., 2005)	Pediatric inpatient in a hospital	Infants aged 3 to 12 months with first episode of wheezing and dyspnea, and RSV detected by ELISA (*N* = 61)	1. Neb. L-epinephrine 1 mg + Neb. Dexamathasone 0.25 mg every 6 h (*n* = 29)	3.3 ± 2.5	3 months	The proportion of in-hospital stay of patients was lower in Group 1 than Group 2 in days 5 and 6 (*p* < 0.05). Follow-up at 3 months did not reveal any significant differences between the groups with respect to hospitalization rates. No significant differences were noted in clinical score, oxygen saturation, duration of supplemental oxygen, and duration IV fluids.
			2. Neb. L-epinephrine 1 mg + Neb. 0.5 ml 0.9% Saline as Placebo every 6 h (*n* = 32)	3.8 ± 2.0		
Turkey (Kuyucu et al., 2004)	Pediatric outpatient clinic and emergency department in a hospital	Infants aged 2 to 21 months with first episode of wheezing, tachypnea, increased respiratory effort, clinical evidence of viral illness such as coryza, fever, and RDAI score =4 (*N* = 69)	1. Single dose of Neb. L-epinephrine 3 mg + I.M. Dexamethasone 0.6 mg/kg (*n* = 23)	7.2 ± 0.8	5 days	None of the patients in any group required hospitalization. No significant differences in heart rate and respiratory rate between groups at 120-min and 24-h after treatment. On 5th day, RDAI score of Group 1 was significantly better than Group 3 and Group 4 (2.3 ± 0.1 vs. 2.9 ± 0.2 and 3.4± 0.2, *p* < 0.05).
			2. Single dose of Neb. Salbutamol 0.15 mg/kg in 0.9% Saline + I.M. Dexamethasone 0.6 mg/kg (*n* = 23)	7.9 ± 1.0		
			3. Single dose of Neb. Epinephrine 3 mg + I.M. Placebo (*n* = 11)	9.6 ± 1.3		
			4. Single dose of Neb. Salbutamol 0.15 mg/kg in 0.9% Saline + I.M. Placebo (*n* = 12)	9.9 ± 1.7		
Paraguay(Mesquita et al., 2009)	Emergency department in a hospital	Infants aged 2 to 24 months with respiratory distress comprising respiratory rate of 40 to 80/min and wheezing, and within 7 days after onset of a cold (*N* = 65)	1. Two doses of Neb. L-epinephrine 1 mg at 30 min apart + Single oral dose of Dexamethasone 0.5 mg/kg (*n* = 33)	7.3 ± 4.0	4 h	Hospitalization rate was similar between groups. There were no significant differences in RDAI score, heart rate, respiratory rate, and oxygen saturation between groups at 1- and 4-h after treatment.
			2. Two doses of Neb. L-epinephrine 1 mg at 30 min apart + Single oral dose of Placebo (*n* = 32)	5.9 ± 3.0		
Canada (Plint et al., 2009)	Eight pediatric emergency departments	Infants aged 6 weeks to 12 months with first episode of wheezing and RDAI score of 4 to 15 (*N* = 800)	1. Two doses of Neb. Racemic epinephrine 3 mg at 30 min apart + Oral Dexamethasone 1 mg/kg as loading, followed by 0.6 mg/kg once daily for 5 days (*n* = 200)	5.0 (3.0–7.0)^*^	7 days and 22 days	By 7th day, Group 1 was significantly less likely than other groups to be hospitalized (RR: 0.65, 95% CI: 0.45 to 0.95, *p* = 0.02). Infants in Group 1 had significantly lower RDAI score (−2.5 ± 2.6 vs. −1.7 ± 2.4, *p* < 0.001) and respiratory rate (−4.0 ± 9.2 vs. −2.9 ± 10.2, *p* < 0.04) during the first hour than placebo.
			2. Two doses of Neb. Racemic epinephrine 3 mg at 30 min apart + Oral Placebo for 5 days (*n* = 199)	5.0 (3.0–7.0)^*^		
			3. Two doses of Neb. Placebo + Oral Dexamethasone 1 mg/kg as loading, followed by 0.6 mg/kg once daily for 5 days (*n* = 200)	5.0 (3.0–7.0)^*^		
			4. Two doses of Neb. Placebo + Oral Placebo for 5 days (*n* = 201)	5.0 (3.0-7.0)^*^		

§ *Values are presented as mean ± standard deviation unless otherwise stated*.

* *Values are median (interquartile range)*.

*RDAI, Respiratory Distress Assessment Instrument; ELISA, Enzyme-Linked Immunosorbent Assay; Neb, Nebulized; I.M., Intramuscular*.

The risk of bias of individual study is presented in Supplementary Material. All studies had most domains judged as low risk of bias. Only one study obtained funding from the pharmaceutical industry (Mesquita et al., 2009).

### Primary outcomes

Four studies examined the impact of epinephrine and dexamethasone combination therapy on hospital admission rate (Kuyucu et al., 2004; Mesquita et al., 2009; Plint et al., 2009; Bawazeer et al., 2014). In the study by Plint et al., the outcome of hospital admission by day 7 and day 22 after enrolment was determined through telephone follow-up and confirmed by chart review. In the study by Mesquita et al., hospital admission was determined at the end of fourth hour of study medication administration. The rate of hospital admission associated with bronchiolitis in the study by Bawazeer et al. was recorded at 4 h, 3 days, and 7 days of the first dose of treatment. Kuyucu et al. documented hospitalization rate within 5 days after intervention.

**Figure 1:**
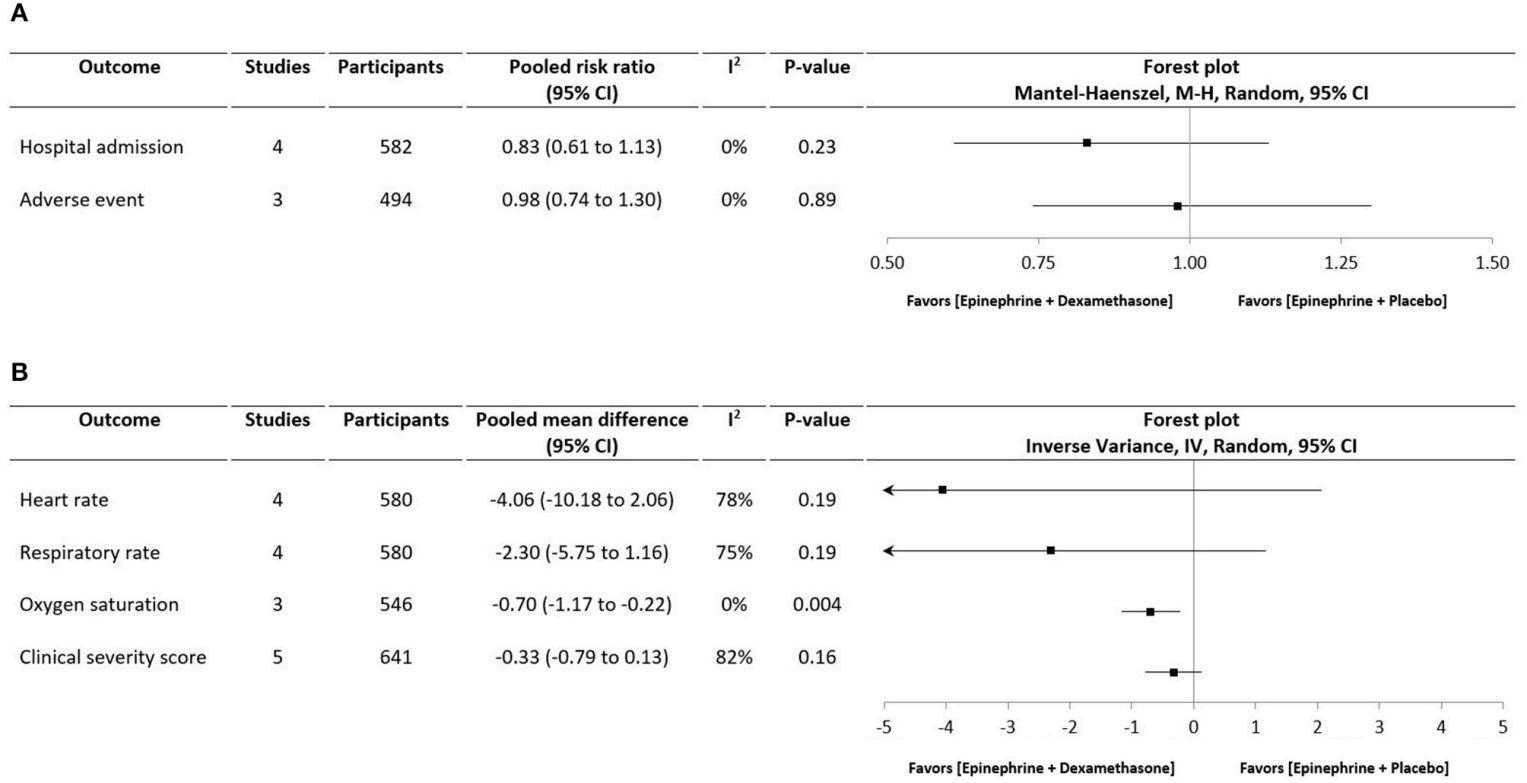
Forest plots showing **(A)** Rate of hospital admission and rate of adverse event; **(B)** Changes in clinical measures of patients treated with combined therapy of epinephrine and dexamethasone vs. epinephrine monotherapy.

Treatment with epinephrine and dexamethasone significantly reduced the hospital admission rate compared with placebo in one study (17.1 vs. 26.4%, RR: 0.65, 95% CI: 0.45 to 0.95, *p* = 0.02) (Plint et al., 2009), while the another three studies reported no differences in hospitalization rate when compared to epinephrine (Kuyucu et al., 2004; Mesquita et al., 2009; Bawazeer et al., 2014), salbutamol (Kuyucu et al., 2004; Bawazeer et al., 2014), or the combination therapy of salbutamol and dexamethasone (Kuyucu et al., 2004; Bawazeer et al., 2014). Pooled data from four studies showed no significant difference in the rate of hospital admission across studies comparing epinephrine and dexamethasone vs. epinephrine alone (RR: 0.83, 95% CI: 0.61 to 1.13, *p* = 0.23, Figure 1).

Plint and colleagues reported the median time to discharge was 0.7 h shorter in patient receiving combination therapy of epinephrine and dexamethasone compared to placebo (*p* = 0.02) (Plint et al., 2009). Bentur et al. reported significant reduction in hospital length of stay in premature infants receiving epinephrine-dexamethasone therapy (MD: −2.60 days, 95% CI: −4.56 to −0.64, *p* = 0.018), whereas no benefit in full-term infants (MD: −0.30 day, 95% CI: −1.35 to 0.75, *p* = 0.57) compared to those receiving epinephrine (Bentur et al., 2005). Meta-analysis was not performed due to the variation in method of reporting.

### Secondary outcomes

All studies reported significant improvements in clinical severity score from baseline in patients treated with epinephrine and dexamethasone combination. Pooled analyses showed no differences in illness severity (SMD: −0.33, 95% CI: −0.79 to 0.13, *p* = 0.16), heart rate (MD: −4.06 beats/min, 95% CI: −10.18 to 2.06, *p* = 0.19), and respiratory rate (MD: −2.30 breaths/min, 95% CI: −5.75 to 1.16, *p* = 0.19) in patients who received combination therapy compared to epinephrine alone. However, combination therapy was found to be more effective than epinephrine in improving oxygen saturation (MD: −0.70%, 95% CI: −1.17 to −0.22, *p* = 0.004).

Adverse events documented in the studies included exercise-induced cough (Kuyucu et al., 2004), wheezing (Kuyucu et al., 2004; Bentur et al., 2005), tremor (Plint et al., 2009), pallor (Plint et al., 2009), vomiting (Plint et al., 2009), dark stools (Plint et al., 2009), hypertension (Plint et al., 2009), and hyperkalemia (Plint et al., 2009). Pooled data suggested no difference in adverse events between both groups (RR: 0.98, 95% CI: 0.74 to 1.30, *p* = 0.89).

## Discussion

There is currently a lack of evidence for effectiveness of different treatment modalities for acute bronchiolitis. The evidence base is even more limited in pediatric populations, due to safety, ethical, and social issues. At present, more than 70% of Canadian emergency department physicians have combined steroid treatment with epinephrine (Plint et al., 2015). Despite the possibility as a treatment that directly addresses inflammatory gene expression in asthma (Florin et al., 2017), results of this study suggest that epinephrine and corticosteroid combination treatment does not provide any clinically important benefit for the treatment of acute bronchiolitis. Some improvements were observed in oxygen saturation level, notwithstanding, the benefit is inconsequential and unlikely to be meaningful for clinicians and patients. Our results are contrary to the benefits previously been highlighted by Hartling et al. (2011b). Findings from our meta-analysis do not support the practice of using combined epinephrine and dexamethasone for acute bronchiolitis, and do provide an argument to reconsider the endorsement of its use in any local or national clinical practice guidelines.

Though several studies and reviews have evaluated the use of bronchodilator medications for viral bronchiolitis, most randomized controlled trials have failed to demonstrate a consistent benefit from alpha or beta-adrenergic agents (Ralston et al., 2014). Several meta-analyses and systematic reviews have depicted that bronchodilators may improve clinical symptom scores, but they do not affect disease resolution, need for hospitalization, or length of stay (Kellner et al., 1996; Flores and Horwitz, 1997; Hartling et al., 2003; King et al., 2004; Wainwright, 2010; Zorc and Hall, 2010). For example, the Cochrane review reported that bronchodilators such as salbutamol do not improve oxygen saturation, hospital admission after outpatient treatment, duration of hospitalization or time to resolution of illness at home (Gadomski and Scribani, 2014). Similarly, another review reported that that the use of epinephrine was ineffective in reducing the post emergency department visit and length of hospital stay (Hartling et al., 2011a).

In the Canadian Bronchiolitis Epinephrine Steroid Trial (CanBEST) conducted by Plint et al., the authors found an unexpected synergism between epinephrine and dexamethasone. Combined therapy with epinephrine and dexamethasone, as compared with placebo, appeared to reduce the rate of hospital admission in the 7 days after study enrolment by 9 percentage points, with a relative risk reduction of 35%. The effects of combining epinephrine and dexamethasone were most apparent in the first 3 days after study enrolment. The investigators also noticed an ostensible benefit from combined therapy on the secondary endpoints, including infants in the combinatorial therapy group were discharged earlier from medical care and resumed quiet breathing and normal feeding sooner than did those in the placebo group. On the contrary, neither dexamethasone alone nor epinephrine alone had any effect on these outcomes (Plint et al., 2009). Although there were no serious short-term adverse events among the infants participated in the study, data from long-term follow-up to establish if the study treatment regimens caused adrenal suppression, arrest of somatic growth, or neurodevelopmental delay were unavailable (Streck and Lockwood, 1979; Zora et al., 1986; Wenning et al., 1994). Albeit the findings are promising with regards to the combinatorial therapy, it has to be reproducible in inpatient environment, compared against placebo, and adverse events need to be judiciously evaluated before the treatment is recommended.

In view of the high burden of disease from bronchiolitis, clinician concerns over corticosteroid use and controversies associated with its management (Plint et al., 2015), we recommend well-designed RCTs which measure the clinical efficacy of combined epinephrine and dexamethasone therapy to be conducted in a standardized manner. The trials should be designed to examine not only the high doses of epinephrine and dexamethasone, but also the lower dose regimen as recommended by drug formularies such as BNF for Children (BNF, 2016) and Lexicomp Pediatric & Neonatal Dosage Handbook (Taketomo et al., 2016). As the decision to institute therapy with corticosteroid necessitates careful consideration of the relative risks and benefits in individual patient, an optimal dose has to be established. Adrenal suppression from exogenous corticosteroid usage remains a risk with short courses of corticosteroid (Streck and Lockwood, 1979; Zora et al., 1986; Wenning et al., 1994). Apprehension has been articulated about plausible developmental delay after treatment with corticosteroid (AAP and CPS, 2002). Corticosteroid therapy may lengthen viral shedding in patients with bronchiolitis (Hall et al., 1986). In consideration of the scarce evidence to be certain of the safety, we suggest future trials to decipher a strategy of minimally dosed prophylactic corticosteroid which would provide significant benefits without any severe adverse events.

A potential weakness of the primary studies was the unavailability of information to delineate the possible role of age, first wheezing episode or further symptom manifestations on response to the combined epinephrine and dexamethasone therapy.

There are several limitations of this review which warrant discussion. One limitation is that only aggregated data is used and hence cannot take into account the patient clinical characteristics or analyze the patients' risk factors within the trials. One study also has a large loss to follow-up compared to the control group (Kuyucu et al., 2004). Most of the trials have a relatively small sample size (Kuyucu et al., 2004; Bentur et al., 2005; Mesquita et al., 2009; Bawazeer et al., 2014). Due to the inclusion of limited number of studies, formal assessment of publication bias is not performed. However, visual inspection of funnel plots suggests little evidence of asymmetry.

Meta-analyses on the outcomes of change in clinical severity score post-treatment, change in heart rate, and change in respiratory rate have revealed considerable heterogeneity (*I*^2^ ≥ 75%) across studies. Subgroup analyses based upon the timing of outcome assessment have successfully diminished the high level of heterogeneity. As such, we presume the heterogeneity may originate from timing of data collection following treatment administration, clinical characteristics of study participants, geographical location, and methods used to conduct the study (random sequence generation).

The present review has also revealed considerable regional discrepancies in the results reported in the individual studies. We have noted that studies reported from North America had a relatively more favorable results compared to those from Middle East, suggesting differences in standard of care as well as ethnicity may play a role in the results obtained.

## Conclusions

Combined treatment of epinephrine and dexamethasone was ineffective in reducing hospital admission and length of stay in infants with bronchiolitis. The therapy appeared to be well-tolerated and pooled data showed some improvements in oxygen saturation favoring the combined therapy. The minimal benefit did not support its use in the treatment of bronchiolitis.

## Author contributions

KK and SL contributed to the conception or design of the work, the acquisition, analysis, and interpretation of data for the work. KK drafted the manuscript and SL revised it critically. SL provided final approval of the version to be published and is the guarantor of the manuscript.

### Conflict of interest statement

The authors declare that the research was conducted in the absence of any commercial or financial relationships that could be construed as a potential conflict of interest. The reviewer NM and handling Editor declared their shared affiliation, and the handling Editor states that the process nevertheless met the standards of a fair and objective review.

## Abbreviations

BNF: British National Formulary
CanBEST: Canadian Bronchiolitis Epinephrine Steroid Trial
CENTRAL: Cochrane Central Register of Controlled Trials
CINAHL: Cumulative Index to Nursing and Allied Health Literature
EMBASE: Excerpta Medica Database
MD: mean difference
MEDLINE: Medical Literature Analysis and Retrieval System Online
PRISMA: Preferred Reporting Items for Systematic review and Meta-Analysis
RCT: randomized controlled trial
RR: risk ratio
RSV: respiratory syncytial virus
SMD: standardized mean difference.
