# Supplementary material for: Systematic Review and Meta-Analysis of the Efficacy and Safety of Combined Epinephrine and Corticosteroid Therapy for Acute Bronchiolitis in Infants
Source: Front Pharmacol. 2017 Jun 22;8:396. doi: 10.3389/fphar.2017.00396 (PMC5479924; doi:10.3389/fphar.2017.00396)
Supplement: Supplementary file 1 [file DataSheet1.DOCX]

Supplementary Material

**Systematic Review and Meta-analysis of the Effectiveness of Combined Epinephrine and Corticosteroid Therapy for Acute Bronchiolitis in Infants**

**Kok Pim Kua^1,2^, Shaun Wen Huey Lee^1*^**

^1^School of Pharmacy, Monash University Malaysia, Jalan Lagoon Selatan, 47500 Bandar Sunway, Selangor Darul Ehsan, Malaysia

^2^Department of Pharmacy, Petaling District Health Office (Ministry of Health Malaysia), No.1, Wisma SAHOCA, Jalan SS6/3A, Kelana Jaya, 47301 Petaling Jaya, Selangor Darul Ehsan, Malaysia

*** Correspondence:**Shaun Wen Huey Lee

Tel: +6 (03) 5514 5890

Fax: +6 (03) 5514 6364

E-mail: [shaun.lee@monash.edu](mailto:shaun.lee@monash.edu)

# Supplementary Material

Database: MEDLINE Search Strategy:

1. bronchodilator

2. bronchodilating agent

3. adrenergic

4. adrenaline

5. adrenalin

6. epinephrine

7. #1 or #2 or #3 or #4 or #5 or #6

8. glucocorticoid

9. corticosteroid

10. steroid

11. anti-inflammatory

12. antiinflammatory

13. betamethasone

14. beclomethasone

15. beclometasone

16. dexamethasone

17. budesonide

18. prednisolone

19. prednisone

20. methylprednisolone

21. methylprednisone

22. meprednisone

23. fluticasone

24. hydrocortisone

25. #8 or #9 or #10 or #11 or #12 or #13 or #14 or #15 or #16 or #17 or #18 or #19 or #20 or #21 or #22 or #23 or #24

26. bronchiolitis

27. respiratory syncytial virus

28. rsv

29. #26 or #27 or #28

30. #7 and #25 and #29; Filters: Publication date to February 28, 2017

Database: EMBASE Search Strategy:

1. bronchodilator.mp.

2. bronchodilating agent/

3. adrenergic.mp.

4. adrenaline.mp.

5. adrenalin/

6. epinephrine.mp.

7. 1 or 2 or 3 or 4 or 5 or 6

8. glucocorticoid/

9. corticosteroid/

10. steroid/

11. anti-inflammatory.mp.

12. antiinflammatory.mp.

13. betamethasone/

14. beclomethasone.mp.

15. beclometasone/

16. dexamethasone/

17. budesonide/

18. prednisolone/

19. prednisone/

20. methylprednisolone/

21. methylprednisone.mp.

22. meprednisone/

23. fluticasone/

24. hydrocortisone/

25. 8 or 9 or 10 or 11 or 12 or 13 or 14 or 15 or 16 or 17 or 18 or 19 or 20 or 21 or 22 or 23 or 24

26. bronchiolitis/

27. respiratory syncytial virus.mp.

28. rsv.mp.

29. 26 or 27 or 28

30. 7 and 25 and 29; Date of Publication: 1974 to 2017 February 28

Database: CINAHL Search Strategy:

1. bronchodilator

2. bronchodilating agent

3. adrenergic

4. adrenaline

5. adrenalin

6. epinephrine

7. S1 or S2 or S3 or S4 or S5 or S6

8. glucocorticoid

9. corticosteroid

10. steroid

11. anti-inflammatory

12. antiinflammatory

13. betamethasone

14. beclomethasone

15. beclometasone

16. dexamethasone

17. budesonide

18. prednisolone

19. prednisone

20. methylprednisolone

21. methylprednisone

22. meprednisone

23. fluticasone

24. hydrocortisone

25. S8 or S9 or S10 or S11 or S12 or S13 or S14 or S15 or S16 or S17 or S18 or S19 or S20 or S21 or S22 or S23 or S24

26. bronchiolitis

27. respiratory syncytial virus

28. rsv

29. S26 or S27 or S28

30. S7 and S25 and S29; Limiters - Published Date: 20170228

Database: CENTRAL Search Strategy:

1. bronchodilator.mp. [mp=title, short title, abstract, full text, keywords, caption text]

2. bronchodilating agent.mp. [mp=title, short title, abstract, full text, keywords, caption text]

3. adrenergic.mp. [mp=title, short title, abstract, full text, keywords, caption text]

4. adrenaline.mp. [mp=title, short title, abstract, full text, keywords, caption text]

5. adrenalin.mp. [mp=title, short title, abstract, full text, keywords, caption text]

6. epinephrine.mp. [mp=title, short title, abstract, full text, keywords, caption text]

7. 1 or 2 or 3 or 4 or 5 or 6

8. glucocorticoid.mp. [mp=title, short title, abstract, full text, keywords, caption text]

9. corticosteroid.mp. [mp=title, short title, abstract, full text, keywords, caption text]

10. steroid.mp. [mp=title, short title, abstract, full text, keywords, caption text]

11. anti-inflammatory.mp. [mp=title, short title, abstract, full text, keywords, caption text]

12. antiinflammatory.mp. [mp=title, short title, abstract, full text, keywords, caption text]

13. betamethasone.mp. [mp=title, short title, abstract, full text, keywords, caption text]

14. beclomethasone.mp. [mp=title, short title, abstract, full text, keywords, caption text]

15. beclometasone.mp. [mp=title, short title, abstract, full text, keywords, caption text]

16. dexamethasone.mp. [mp=title, short title, abstract, full text, keywords, caption text]

17. budesonide.mp. [mp=title, short title, abstract, full text, keywords, caption text]

18. prednisolone.mp. [mp=title, short title, abstract, full text, keywords, caption text]

19. prednisone.mp. [mp=title, short title, abstract, full text, keywords, caption text]

20. methylprednisolone.mp. [mp=title, short title, abstract, full text, keywords, caption text]

21. methylprednisone.mp. [mp=title, short title, abstract, full text, keywords, caption text]

22. meprednisone.mp. [mp=title, short title, abstract, full text, keywords, caption text]

23. fluticasone.mp. [mp=title, short title, abstract, full text, keywords, caption text]

24. hydrocortisone.mp. [mp=title, short title, abstract, full text, keywords, caption text]

25. 8 or 9 or 10 or 11 or 12 or 13 or 14 or 15 or 16 or 17 or 18 or 19 or 20 or 21 or 22 or 23 or 24

26. bronchiolitis.mp. [mp=title, short title, abstract, full text, keywords, caption text]

27. respiratory syncytial virus.mp. [mp=title, short title, abstract, full text, keywords, caption text]

28. rsv.mp. [mp=title, short title, abstract, full text, keywords, caption text]

29. 26 or 27 or 28

30. 7 and 25 and 29; Search Limits: - Published Date: 2017 February 28

# Supplementary Figures and Tables

## Supplementary Table 1: List of full-text articles excluded from evidence synthesis

| **No.** | **Study** | **Title** | **Reason for exclusion** |
| --- | --- | --- | --- |
| 1. | Ahronheim S. (2010) | Combination therapy with epinephrine and dexamethasone for bronchiolitis | Review |
| 2. | Alansari K., et al. (2013) | Oral dexamethasone for bronchiolitis: a randomized trial | No epinephrine |
| 3. | Al-Shawwa B., et al. (2007) | Clinical and therapeutic variables influencing hospitalisation for bronchiolitis in a community-based paediatric group practice | Medical record review |
| 4. | Babl F.E., et al. (2008) | Bronchiolitis management in pediatric emergency departments in Australia and New Zealand: a predict study | Survey |
| 5. | Barlas C., et al. (1998) | Racemic adrenaline and other treatment regimens in mild and moderate bronchiolitis | Intervention lacked combination therapy |
| 6. | Beck R., et al. (2007) | Computerized acoustic assessment of treatment efficacy of nebulized epinephrine and albuterol in RSV bronchiolitis | Intervention lacked combination therapy |
| 7. | Berger I., et al. (1998) | Efficacy of corticosteroids in acute bronchiolitis: short-term and long- term follow-up | Intervention lacked combination therapy |
| 8. | Blom D.J.M., et al. (2007) | Inhaled corticosteroids during acute bronchiolitis in the prevention of post-bronchiolitic wheezing | Review |
| 9. | BMJ (2011) | Steroids and bronchodilators for acute bronchiolitis in babies and toddlers | Brief item |
| 10. | Cade A., et al. (2000) | Randomised placebo controlled trial of nebulised corticosteroids in acute respiratory syncytial viral bronchiolitis | Intervention lacked combination therapy |
| 11. | Didion B., et al. (2012) | What are the most effective therapies for bronchiolitis? | Evidence-based answer |
| 12. | Ducharme F.M., et al. (2011) | Management of acute bronchiolitis: Inhaled adrenaline shows promise in outpatients, but treatment for inpatients remains unclear | Editorial |
| 13. | Fernandes R.M., et al. (2013) | Glucocorticoids for acute viral bronchiolitis in infants and young children | Review |
| 14. | Fiandeiro P.T. (2009) | Epinephrine and dexamethasone reduce hospital admission in children with bronchiolitis | Correspondence |
| 15. | Florin T.A., et al. (2017) | Viral bronchiolitis | Review |
| 16. | Frohna J.G., et al. (2009) | Combination of epinephrine and dexamethasone may reduce hospitalization in children with bronchiolitis | Commentary |
| 17. | Ghanei M., et al. (2007) | Inhaled corticosteroids and long-acting beta 2-agonists in treatment of patients with chronic bronchiolitis following exposure to sulfur mustard | No pediatric patient |
| 18. | Gómez-y-López R.E., et al. (2007) | Comparative clinical study of dexamethasone vs. nebulized salbutamol in acute bronchiolitis | Intervention lacked combination therapy |
| 19. | Hartling L., et al. (2011) | Epinephrine for bronchiolitis | Review |
| 20. | Hartling L., et al. (2011) | Steroids and bronchodilators for acute bronchiolitis in the first two years of life: systematic review and meta-analysis | Review |
| 21. | Jendi M.R., et al. (2016) | Do corticosteroids reduce bronchiolitis hospitalizations? | Evidence-based answer |
| 22. | Kajosaari M., et al. (2000) | Inhaled corticosteroids during and after respiratory syncytial virus-bronchiolitis may decrease subsequent asthma | Intervention lacked combination therapy |
| 23. | Karaatmaca B., et al. (2010) | Comparison of the effectiveness of nebulized budesonide, salbutamol and adrenaline in the treatment of acute bronchiolitis: a randomized double blind placebo controlled clinical trial | Intervention lacked combination therapy |
| 24. | Klassen T.P., et al. (1997) | Dexamethasone in salbutamol-treated inpatients with acute bronchiolitis: a randomized, controlled trial | No epinephrine |
| 25. | Klassen T.P., et al. (2008) | Dexamethasone and placebo did not differ for respiratory status change or hospital admission in infants with acute bronchiolitis | Commentary |
| 26. | Labbé A., et al. (1996) | Treatment of acute bronchiolitis in infants. Role of bronchodilators and steroids | Review |
| 27. | McBride J.T. (2002) | Dexamethasone and bronchiolitis: a new look at an old therapy? | Editorial |
| 28. | McCulloh R.J., et al. (2012) | Hospitalist and nonhospitalist adherence to evidence-based quality metrics for bronchiolitis | Retrospective chart review |
| 29. | Milner A.D. (1997) | The role of corticosteroids in bronchiolitis and croup | Review |
| 30. | Norman B.C., et al. (2011) | Fluticasone, azithromycin and montelukast therapy in reducing corticosteroid exposure in bronchiolitis obliterans syndrome after allogeneic hematopoietic SCT: a case series of eight patients | Case series |
| 31. | Ochoa Sangrador C., et al. (2014) | Overuse of bronchodilators and steroids in bronchiolitis of different severity. Bronchiolitis-study of variability, appropriateness, and adequacy | Cross-sectional study |
| 32. | Patel H., et al. (2003) | Randomized, double-blind, placebo-controlled trial of oral albuterol in infants with mild-to-moderate acute viral bronchiolitis | Intervention lacked combination therapy |
| 33. | Perreaux F. (2006) | Dexamethasone inhalations in RSV bronchiolitis: a double-blind, placebo-controlled study | Note |
| 34. | Pinto J.M., et al. (2016) | Duration of hospitalization in association with type of inhalation therapy used | Retrospective chart review |
| 35. | Plint A.C., et al. (2004) | Practice variation among pediatric emergency departments in the treatment of bronchiolitis | Prospective consecutive cohort study |
| 36. | Plint A.C., et al. (2013) | The current use of bronchodilators and steroids for the management of bronchiolitis in Canadian pediatric emergency departments | Conference abstract |
| 37. | Plint A.C., et al. (2015) | Bronchodilator and steroid use for the management of bronchiolitis in Canadian pediatric emergency departments | Electronic survey |
| 38. | Plint A.C., et al. (2016) | Management of bronchiolitis in community hospitals in Ontario: a multicentre cohort study | Retrospective chart review |
| 39. | Ralston S., et al. (2012) | Epinephrine for acute bronchiolitis, but not steroids alone, reduces hospital admissions | Commentary |
| 40. | Rochat I., et al. (2013) | Pediatrics. New treatment options for viral bronchiolitis | Review |
| 41. | Sakulchit T., et al. (2016) | Nebulized epinephrine for young children with bronchiolitis | Review |
| 42. | Schiltz P., et al. (2011) | What is the status of steroids in the treatment of infants with bronchiolitis? | Review |
| 43. | Schroeder A.R., et al. (2014) | Recent evidence on the management of bronchiolitis | Review |
| 44. | Schuh S., et al. (2008) | A single versus multiple doses of dexamethasone in infants wheezing for the first time | Intervention lacked combination therapy |
| 45. | Singam R., et al. (2006) | Combined fluticasone propionate and salmeterol reduces RSV infection more effectively than either of them alone in allergen-sensitized mice | Animal study |
| 46. | Smith D.K., et al. (2017) | Respiratory syncytial virus bronchiolitis in children | Review |
| 47. | Springer C., et al. (1990) | Corticosteroids do not affect the clinical or physiological status of infants with bronchiolitis | No epinephrine |
| 48. | Sumner A., et al. (2010) | Cost-effectiveness of epinephrine and dexamethasone in children with bronchiolitis | Identical study as Plint A.C., et al. (2013) |
| 49. | Tagarro A., et al. (2014) | Dexamethasone does not reduce length of hospitalization or recurrent wheezing 1 year after early bronchiolitis | Prospective-retrospective observational study |
| 50. | Teeratakulpisarn J., et al. (2007) | Efficacy of dexamethasone injection for acute bronchiolitis in hospitalized children: a randomized, double-blind, placebo-controlled trial | Reporting of results not stratified to combination therapy |
| 51. | Tudor G.J., et al. (2013) | In infants younger than 24 months old and with bronchiolitis, does nebulized epinephrine improve clinical status? | Review |
| 52. | Van Bever H.P., et al. (1996) | Corticosteroids for bronchiolitis | Letter to the editor |
| 53. | Van Dellen Q.M., et al. (2011) | Does combined oral dexamethasone and epinephrine inhalation help infants with bronchiolitis to recover faster? | Commentary |
| 54. | Vandini S., et al. (2016) | Latest options for treatment of bronchiolitis in infants | Review |
| 55. | Veerappan A., et al. (1996) | Role of steroids in croup and beta agonists in bronchiolitis | Review |
| 56. | Wong J.Y.W., et al. (2000) | No objective benefit from steroids inhaled via a spacer in infants recovering from bronchiolitis | No epinephrine |
| 57. | Wright R.B., et al. (2002) | New approaches to respiratory infections in children: bronchiolitis and croup | Review |
| 58. | Zorc J.J., et al. (2010) | Bronchiolitis: recent evidence on diagnosis and management | Review |

## Supplementary Figures


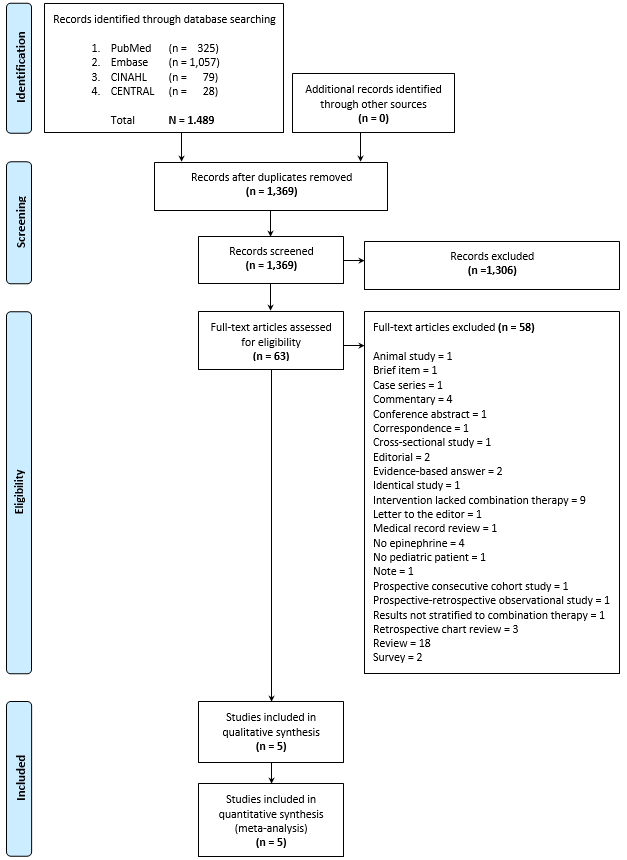


**Supplementary Figure 1.** Preferred Reporting Items for Systematic Reviews and Meta-analyses (PRISMA) flow diagram showing selection of relevant studies for inclusion.


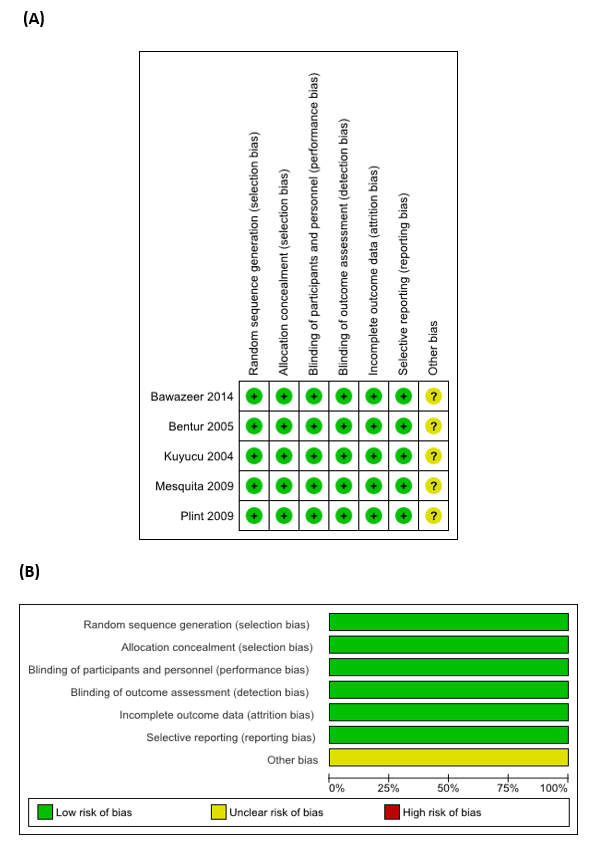


**Supplementary Figure 2.** Assessment of risk of bias according to a recommended tool for randomized controlled trials by the Cochrane Handbook for Systematic Reviews of Interventions. (A) Risk of bias summary showing review authors’ judgments about each risk of bias domain for five randomized controlled trials; (B) Risk of bias graph showing each risk of bias domain presented as percentages across the studies.


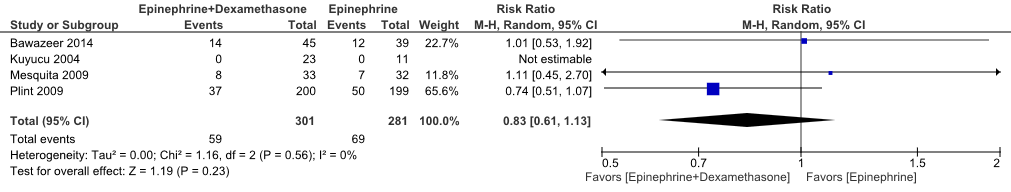


**Supplementary Figure 3.** Forest plot showing the effect of combined therapy with epinephrine and dexamethasone for infants with bronchiolitis on hospital admission rate compared to epinephrine monotherapy.


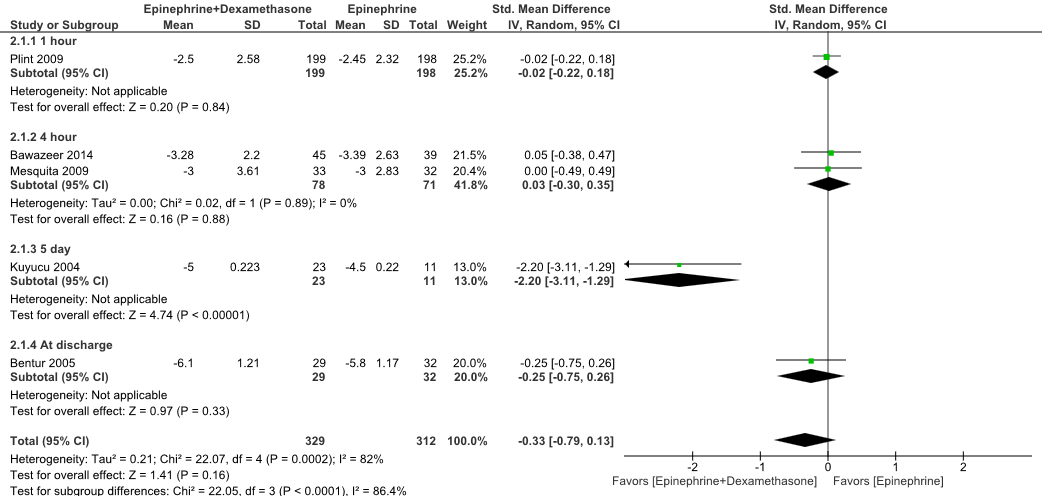


**Supplementary Figure 4.** Forest plot showing the effect of combined therapy with epinephrine and dexamethasone for infants with bronchiolitis on change in clinical severity score post-therapy compared to epinephrine monotherapy.


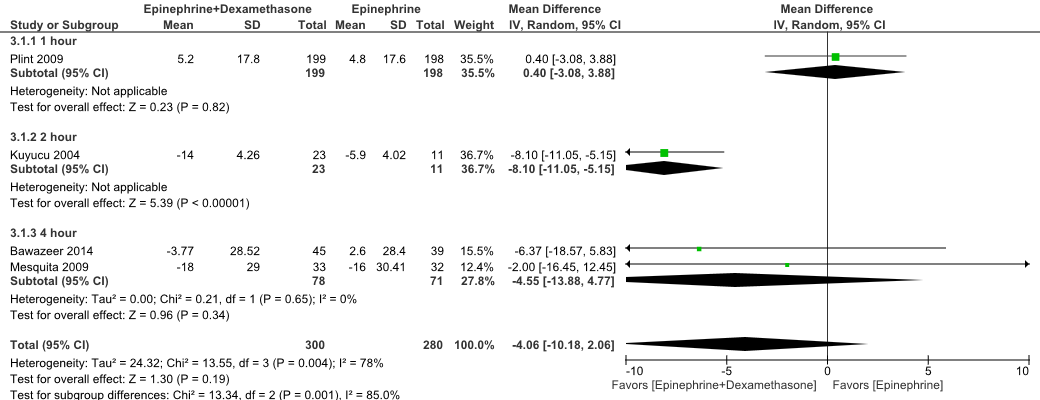


**Supplementary Figure 5.** Forest plot showing the effect of combined therapy with epinephrine and dexamethasone for infants with bronchiolitis on change in heart rate (beats/min) at 1, 2, and 4 hours post-therapy compared to epinephrine monotherapy.


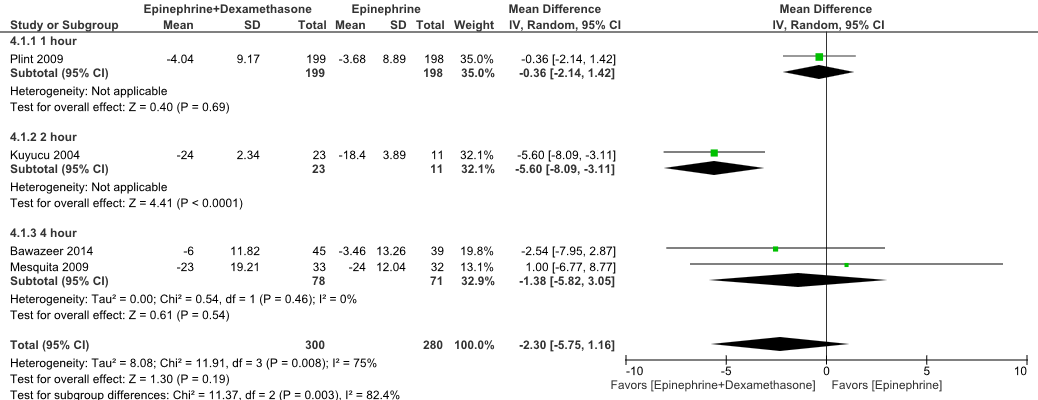


**Supplementary Figure 6.** Forest plot showing the effect of combined therapy with epinephrine and dexamethasone for infants with bronchiolitis on change in respiratory rate (breaths/min) at 1, 2, and 4 hours post-therapy compared to epinephrine monotherapy.


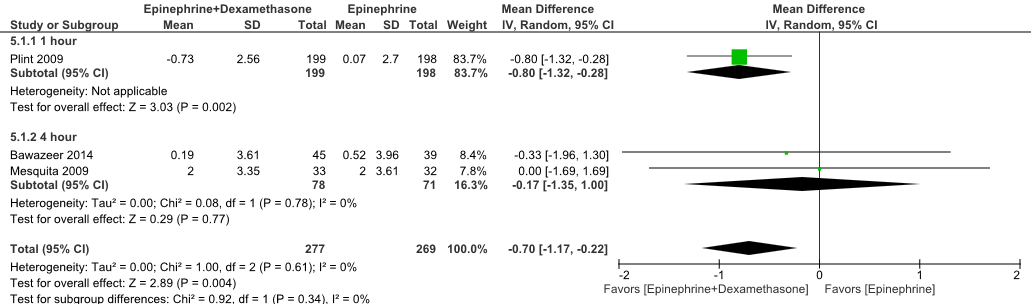


**Supplementary Figure 7.** Forest plot showing the effect of combined therapy with epinephrine and dexamethasone for infants with bronchiolitis on change in oxygen saturation (%) at 1 hour post-therapy compared to epinephrine monotherapy.


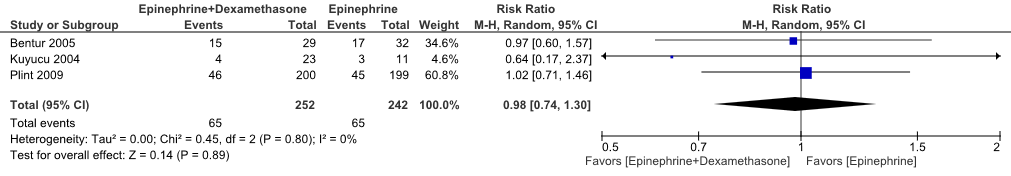


**Supplementary Figure 8.** Forest plot showing the effect of combined therapy with epinephrine and dexamethasone for infants with bronchiolitis on adverse event compared to epinephrine monotherapy.
